# Asfarviruses and Closely Related Giant Viruses

**DOI:** 10.3390/v15041015

**Published:** 2023-04-20

**Authors:** Sihem Hannat, Bernard La Scola, Julien Andreani, Sarah Aherfi

**Affiliations:** 1 Institut Hospitalo-Universitaire Méditerranée Infection, 13005 Marseille, France; 2 MEPHI, Institut de Recherche pour le Développement (IRD), Aix-Marseille Université, 13005 Marseille, France; 3 Assistance Publique des Hôpitaux de Marseille (AP-HM), 13005 Marseille, France; 4 CHU Grenoble Alpes, 27 Boulevard de la Chantourne, 38700 La Tronche, France

**Keywords:** *Asfarviridae*, Faustovirus, Pacmanvirus, Kaumoebavirus, Nucleo-Cytoplasmic Large DNA Viruses (NCLDVs), *Nucleocytoviricota*

## Abstract

*Acanthamoeba polyphaga mimivirus*, so called because of its “mimicking microbe”, was discovered in 2003 and was the founding member of the first family of giant viruses isolated from amoeba. These giant viruses, present in various environments, have opened up a previously unexplored field of virology. Since 2003, many other giant viruses have been isolated, founding new families and taxonomical groups. These include a new giant virus which was isolated in 2015, the result of the first co-culture on *Vermamoeba vermiformis*. This new giant virus was named “Faustovirus”. Its closest known relative at that time was African Swine Fever Virus. Pacmanvirus and Kaumoebavirus were subsequently discovered, exhibiting phylogenetic clustering with the two previous viruses and forming a new group with a putative common ancestor. In this study, we aimed to summarise the main features of the members of this group of giant viruses, including Abalone Asfarvirus, African Swine Fever Virus, Faustovirus, Pacmanvirus, and Kaumoebavirus.

## 1. Introduction

The serendipitous discovery of giant viruses of amoebas broadened the field of virology in a sudden and spectacular way [1], due to their complex genetic and structural characteristics. These viruses are characterised by a particle size of up to 200 nm, similar to some bacteria sizes, making them visible under light microscopy. They have a double-stranded DNA genome and were classified into the group of Nucleo-Cytoplasmic Large DNA Viruses (NCLDVs) [2], at that time classified within the phylum *Nucleocytoviricota* [3]. This phylum constitutes a monophyletic group and shares an ancient common origin [4]. Historically, NCLDVs include the following five families: *Phycodnaviridae*, *Asfarviridae*, *Poxviridae*, *Iridoviridae*, and *Ascoviridae*. Their broad host range includes algae for Phycodnaviruses [5], mainly pigs and boars for Asfarviruses [6], insects for Irido-Ascoviruses [7] and Entomopoxviruses, and rodents, cattle, or humans for Chordopoxviruses [8]. African Swine Fever Virus (ASFV) is still the only known double-stranded DNA (dsDNA) arbovirus transmitted by ticks. It was first described in 1921 by Montgomery [9], and caused haemorrhagic fever in pigs, with very high mortality rates approaching 100% [10]. In 2009, a partial DNA virus was sequenced in a bivalve dinoflagellate *Heterocapsa circularisquama* [11], revealing close phylogenetic proximity with ASFV. In 2015, the use of *Vermamoeba vermiformis* as a novel cell support for the co-culture of giant viruses made it possible to isolate *Faustovirus*, a new virus closely related to ASFV [12]. In 2016, *Kaumoebavirus*, a phylogenetically close relative of *Faustovirus*, was isolated from wastewater collected in Jeddah, Saudi Arabia [13]. In 2017, the first *Pacmanvirus* strain A23 was isolated from sewage collected in Oran, Algeria by co-culture with *Acanthamoeba castellanii.* This virus, which has very different morphological features, phylogenetically clusters with the pre-cited extended clade of *Asfarviridae* [14].

In this study, we aimed to describe the main features of the members of the extended clade of Asfarviruses, including the close relatives isolated by co-culture of protist amoebae and discovered more recently such as Faustoviruses [12], *Kaumoebavirus* [13], Pacmanviruses [14], Abalone Asfarvirus [15], and Metagenome-Assembled Genomes (MAGs).

## 2. African Swine Fever Virus (ASFV)

### 2.1. History of Discovery

This virus has long been classified as an Iridovirus [2,16]. However, some unique features of its structure, as well as the examination of its genome, led the International Committee on Taxonomy of Viruses (ICTV) to create the family *Asfarviridae* (African Swine Fever and relatives) in which the Asfivirus is the only genus recognised and ASFV is the only member [17]. ASFV was discovered in June 1910 in sub-Saharan Africa [9]. In East Africa, a sylvatic cycle was subsequently identified between juvenile warthogs (with ASFV viremia) and soft ticks where the virus can replicate and be transmitted vertically. It then spread to Europe (first outbreak in Lisbon in 1957), China and other parts of Asia, and to South America and the Caribbean (1970s) [18,19,20,21,22], as international cross-border trade developed [23,24] where it became endemic as in Spain and in Portugal. Outbreaks in endemic areas appear to be related to soft ticks, but pig-to-pig contamination appears to be the main current source of transmission.

ASFV is the causative agent of African swine fever, a haemorrhagic syndrome with a mortality rate close to 100% in its acute forms in pigs, warthogs, and wild boars of all age groups [25] in Europe and America [26,27]. It is an arbovirus transmitted by soft ticks from *Ornithodoros* that also constitute a viral reservoir [28,29,30]. It is not transmissible to humans [31]. In its highly virulent forms, ASFV is characterised by high fever, loss of appetite, diarrhoea, cyanosis and generalised haemorrhages, coagulation disorders, and death within an average of between two and ten days [32,33]. ASFV multiplies in monocytes and macrophages [25,34,35] and transmission of the virus to healthy animals occurs through direct (oro-nasal) contact with infected animals, ingestion of contaminated animal by-products, or indirectly through contaminated surfaces (fomite transmission), and feed [33,36,37,38,39]. The high case fatality rate and lack of an efficient vaccine [40] against this virus make ASFV an important threat to worldwide pork production with a potentially major economic impact on the swine farming industry. Furthermore, the legal or illegal importation of pork products has contributed towards the introduction of the virus to new regions and, over the past decade, the virus has emerged in a few countries such as China [41,42], which is a major consumer of pig products, followed by Belgium and Germany [43,44]. ASFV is listed as a “List A disease” according to the World Organisation for Animal Health (WOAH), i.e., its causative agent is classified as a devastating transmissible animal pathogen with a potential rapid spread [45,46].

### 2.2. Structural Features

The icosahedral virions are about 250 nm in diameter [47,48] and encompass a large double-stranded DNA genome [49]. African swine fever virions have a complex multi-layered structure [50]. The capsid results from the assembly of between 1892 and 2172 hexagonal capsomers to form a mature viral particle [49,50,51,52]. Recent high-magnification reconstructions using three complementary techniques and different teams found one major protein (p72) and four other proteins (M1249L, p17, p49 and H240R) involved in the formation of the icosahedral capsid [53,54,55]. This capsid has the same triangulation as Faustovirus (see above) and Medusavirus [56] T = 277 (h = 7, k = 12) (Table 1). Five different layers have now been well-identified from the inside to the outside: the genome forming the nucleoid of 80 nm surrounded by a first thick layer (1st layer), an internal protein core shell with a diameter of 180 nm (2nd layer), an internal lipid membrane (3rd layer), the 250 nm capsid (4th layer) and wrapped in an external envelope for extracellular virions (5th layer) [53,54]. Finally, the virus particle size is 260 nm, including the outer membrane. A recent study identified a transmembrane protein, pE84R, that plays a major role in the assembly of the inner core shell in interaction with polyproteins pp220 and pp62 [57]. ASFV is the only virus of this group with a described outer envelope produced by the host membrane and involved in the entry of the virus into cells (see Section 2.4). This membrane probably explains the lack of efficacy of vaccines producing antibodies against p72 capsid homologs. The detection of homologs from the ultrastructure of ASFV shows closer proximity to the Abalone asfarvirus and Elysia marginata virus (Table 1, H240R, E199L and pA104R homologs) compared to Faustovirus, Kaumoebavirus, and Pacmanvirus.

### 2.3. Phylogenetic and Genomic Features

The ASFV genome consists of a linear double-stranded DNA molecule with a length of approximately 170–194 kb predicting to encode 150–167 Open Reading Frames (ORFs), depending on the isolate. The GC content of the ASFV genome is around 39% [49,51,52,58].

### 2.4. Replication Cycle

ASFV multiplies in the monocytes and macrophages in the blood and bone marrow of pigs. Viral replication has also been observed in endothelial cells, hepatocytes, renal tubular epithelial cells [59,60,61,62], neutrophilic granulocytes [63], megakaryocytic cells [25], thymus epithelial cells, fibroblastic cells, and smooth muscle cells of venules and arterioles [62]. The infectious cycle begins with its adsorption and entry into the host cell. Entry may occur by clathrin-mediated and dynamin-dependent endocytosis, and macropinocytosis [64,65,66,67]. The virus unwraps via an endosomal pathway, then engages the ubiquitin proteasome system for capsid disassembly to release its DNA and initiate replication in the host cytoplasm [68]. Viral factories form in the perinuclear area, sheltering DNA replication and the assembly of neovirions which are ejected from the viral factory with kinesin and, subsequently, from the host cell by budding [69].

### 2.5. Pathogenicity

Asfarviruses represent the only known DNA virus transmitted by arthropods [6]. In Africa, the sylvatic cycle involves warthogs (*Phacochoerus africanus*) and soft ticks belonging to the genus *Ornithodoros*. Juvenile warthogs infected by soft ticks are viraemic, but do not develop any disease and constitute the source of infection for naive ticks taking a blood meal. In Europe, transmission of the virus is usually directly from a sick pig to a healthy pig or indirectly via a mechanical vector such as soiled premises, vehicles, instruments, or clothing [22,37], but can only be transmitted by air over short distances (not exceeding two metres) [70]. Several studies on the acute infection of pigs with ASFV have shown that the main route of viral entry is classically the pharyngeal mucosa and tonsils [25,71]. Transmission to European and Asian wild boars and domestic pigs via infected ticks may not be the main route of infection. Ticks are infectious for up to eight years and can remain without feeding in a quiescent state for up to five years [72,73]. They can live for several years, and one of the basic characteristics of ASFV is that it can persist for several weeks or months in frozen or uncooked meat [17].

The virus then passes into the retropharyngeal lymph nodes, whence it disseminates into the blood. The incubation period of the disease may vary between 4 and 19 days [30]. Clinical presentations are diverse, ranging from chronic to high acute infection, to death. The pathogenesis of ASFV is explained, in the early stages of infection, by the massive destruction of monocytes and macrophages releasing active products that disrupt haemostasis [32].

The main manifestations of the infection are thrombocytopaenia, leukopaenia, lymphopaenia, [25] and haemorrhages, leading to death [74,75].

## 3. Faustovirus

### 3.1. History of Discovery

Historically, only the genus *Acanthamoeba* was used for giant virus isolation. In 2015, the implementation of a high throughput culture strategy by diversifying the cell supports made it possible to isolate a new giant virus: Faustovirus E12 [12]. This was the first giant virus of amoebas isolated by co-culture on *Vermamoeba vermiformis*, which is reported to be the most common free-living protist in human environments [76,77,78]. This new co-culture approach quickly enabled the isolation of many other giant viruses, including Faustovirus mariensis [79] and 16 other strains of Faustoviruses [13,80,81,82,83]. Faustoviruses were all isolated from wastewater samples collected in France, Senegal, Lebanon and Saudi Arabia [12]. For example, the ST1 strain was isolated from wastewater collected in Saint-Pierre-de-Mézoargues, France and the LC9 strain was collected from sewage in La Ciotat, France [84].

### 3.2. Structural Features

Faustoviruses virions harbour icosahedral symmetry and have a diameter of about 250 nm, as determined by cryo-electron microscopy [12,85]. Faustoviruses are unique among DNA viruses in that they harbour an imbricated double protein shell that encapsidates its genome. The outer shell is composed of a double jelly-roll fold protein, a feature shared with numerous double-stranded DNA viruses, encoded by a gene with a very unusual organisation, containing a great number of introns and exons, differing from other known viral capsid structures [86]. Moreover, the inner capsid shell consists in a repeated hexameric unit, which is very flexible and quasi-icosahedral.

### 3.3. Phylogenetic and Genomic Features

By the start of 2023, 18 complete genomes of Faustoviruses had been published. Genome lengths range from 455,803 bp (for Faustovirus E3) to 491,024 bp (for Faustovirus E9) [87,88]. The GC content varies from 36.22% to 39.59%, which is in the same range order as those of *Asfarviridae* members (31–45%) [87,89]. Their genomes are probably linear [88] and do not encode any tRNA. Interestingly, for Faustovirus, the unique structure of the major capsid protein and its encoding gene have been exhaustively studied for the strain E12 [86]. The Faustovirus major capsid protein gene is dispersed over a 17 kbp region, while coding for a protein which is 645 amino acids in length, resulting from an extensive splicing of 13 exons [12,86,90] separated by 12 introns for Faustovirus E12. These introns are characterised by a mean length of 1273 bp, which is larger than that previously described in viruses and a mean GC content of 35.2%, while exons have a shorter mean length of 149 bp and a higher mean GC content of 43.9%. Transcriptomic study revealed that during the first three hours of the replication cycle in amoeba, the intronic regions are transcribed while the exonic regions are not. The exonic region transcripts begin to be detected six hours post-infection, with a maximum transcription rate 11 h post-infection, while transcripts of intronic regions significantly decrease. The introns detected in the Faustovirus E12 major capsid protein gene are, surprisingly, of two types: group I introns that can self-splice, and spliceosomal introns that use non-canonical splice-sites in their excision.

Genome-wide alignments of Faustoviruses revealed a high level of co-linearity, but an increased indel accumulation and local rearrangements have been noted more frequently in the Major Capsid Protein gene region and the extremities of the genomes, revealing a faster rate of evolution. In addition, most Faustoviruses have terminal inverted repeats (TIRs) at both ends of their genome [86], so the presence of these repeats is similar in ASFV [91] and other large DNA viruses such as Phycodnavirus [92] or Poxviruses [93].

Phylogenetic analyses based on the core genes, especially the DNA polymerase B, show that Faustovirus is a distant relative of Asfarviridae [87]. A more deeply phylogenetic analysis based on 267 single-copy core genes revealed three main clades among Faustoviruses, namely E9, D, and M/L [88]. This clustering showed congruency with the high intra-clade level of nucleotide similarity for these core genes, ranging between 92% and 100%, while it ranges from 64.5% and 70.5% for interclade similarity.

### 3.4. Replication Cycle

The entire Faustovirus replication cycle in *V. vermiformis* lasts 18–20 h, which is slightly shorter than that of Mimivirus (14 h) [12]. At the beginning of the virus replication cycle, between two and four hours’ post-infection, Faustovirus virions are located in host phagosomes, close to the amoeba nucleus. The eclipse phase lasts between 4 and 6 h post-infection, which is longer than for Mimiviruses. Viral factories then form, which are visible close to the host nucleus and are surrounded by mitochondria. Between 16 and 18 h, they fill the host cytoplasm and are filled with neovirions. New viral particles are released by cell lysis around 18 to 20 h post-infection [12].

### 3.5. Pathogenicity

*Vermamoeba vermiformis* is, to date, the only host of Faustovirus [94]. This highly prevalent amoeba is ubiquitous, and often associated with human water environments. Due to its thermotolerance, it is able to colonise hot water systems and can be the reservoir of bacteria. However, to date, there is no evidence of any real impact of Faustovirus on human health, although metagenomic studies sequencing small PCR products have detected positive sequences related to Faustovirus DNA polymerase in between 9.82% and 11.76% of sera collected from febrile and healthy humans in Senegal [85]. Faustovirus-like sequences have also been identified in metagenomes obtained from arthropods and rodents, although the link between Faustoviruses-like sequences and arthropods or rodents remains unknown and is deserving of further study.

## 4. Kaumoebavirus

### 4.1. History of Discovery

Kaumoebaviruses are the second giant viruses isolated by co-culture on *V. vermiformis*. The strain Kaumoebavirus KV-Sc [13] was the first to be isolated from an environmental wastewater sample collected in the southern region of Jeddah in Saudi Arabia. Its name comes from King Abdulaziz University Amoeba. A second strain, Kaumoebavirus-LCC10 (KV-LCC10), was isolated from a wastewater sample collected in La Ciotat, in Southeastern France [95].

### 4.2. Structural Features

Kaumoebavirus virions are icosahedral particles of ∼250–260 nm in diameter, devoid of fibrils.

### 4.3. Genomic and Phylogenetic Features

The Kaumoebavirus genome consists of a 350 kbp to 362.6 kbp double-stranded DNA linear molecule, which is shorter than those of Faustoviruses, with a GC content ranging from 43.1% to 43.7% [13,95]. They do not encode any tRNA. For Kaumoebavirus LCC10, it has been shown that genome extremities consist in 1032 bp-long Inverted Terminal Repeats, flanked by closed hairpin termini of 102 bp in length [95].

The genomes of two strains both encode for 465 genes and are collinear for the main part of their length, albeit with a total of 180 ORFs detected as lost or gained in one or other genome. A total of 59% of Kaumoebavirus KV-Sc proteins are homologous with other viral proteins, including 43% with Faustovirus and 23% with Asfarviruses. It is noteworthy that 67–76% of their gene content are ORFans. Although Faustoviruses are the closest relatives, genome co-linearity is not conserved with Kaumoebavirus KV-Sc [13,95]. As for *Asfarviridae* members and Faustoviruses, A/T rich motifs are abundant, widely distributed upstream to start codons, especially upstream to the core genes of Asfarviruses, Faustoviruses, and Kaumoebavirus, and play an important role as promoter sequences [96]. Notably, the Kaumoebavirus genomes harbour great gene strand bias, with a large majority of genes encoded by the positive strand along about two-thirds of the genome length. Phylogenetic reconstructions show that Kaumoebaviruses cluster together with Faustoviruses and the genus Asfivirus, suggesting that they may compose a new genus member among the family *Asfarviridae*. Interestingly, the region predicted to encode the MCP shows intron differences between the Sc and LCC10 strains. However, and as is the case in Faustovirus, this region contains ORFs encoding a GIY-YIG endonuclease. This specific region is in the middle of the genome and seems to have a higher rate of evolution.

### 4.4. Replication Cycle

The replication cycle of KV-Sc lasts between 16 and 20 hours. Kaumoebavirus particles enter into *Vermamoeba vermiformis* through phagocytosis. At three hours post-infection, virions are often visualised as clusters of two to four particles in vacuoles. The “eclipse” phase occurs at four hours post-infection, followed by the formation of viral factories at six hours post-infection. New Kaumoebavirus particles fill viral factories until 16 h post-infection and are released at 20 h post-infection through cell lysis. The nuclear morphology of the host cells is not modified during the replication cycle of Kaumoebavirus, in contrast to what happens with Faustovirus [13].

## 5. Pacmanvirus

### 5.1. History of Discovery

Pacmanvirus A23 was isolated from a water sample collected from a well on a roadside in Algeria (North Africa) [14], by co-culture of *A. castellanii* [97,98]. The name “Pacmanvirus” was given with respect to the shape of the irregular icosahedral capsid, with some aspects of broken capsid observed in negative staining micrographs [14]. A second strain, named Pacmanvirus S19, was isolated from wastewater samples from Algeria in 2021, using the same amoebal host, *Acanthamoeba castellanii* [99]. A third strain and its genome are now available, *Pacmanvirus lupus*, isolated from a Siberian *Canis lupus* intestine collected in permafrost (dated at over 27,000 years) [100].

### 5.2. Structural Features

Pacmanvirus virions are icosahedral in shape with an overall external structure close to that of Faustovirus, of about 250 nm in diameter. The major capsid protein of Pacmanvirus is double jelly-roll fold, as confirmed by three-dimensional cryo-EM reconstruction, and it has an inner membrane (IMV) but no distinct inner capsid shell [14]. The capsid is encoded by a single ORF, like those of ASFV, but at the protein level, it is more similar to those of Faustovirus. In addition, genome analysis contributes towards identifying a predicted minor capsid protein in Pacmanvirus S19, lupus and A23.

### 5.3. Genomic and Phylogenetic Features

The complete genome of Pacmanvirus A23 is linear shaped and reaches 395,405 bp in length, which is shorter than Faustoviruses and longer than ASFV and Kaumoebaviruses. It encodes for 465 genes with a GC content of 33.6%, with the particularity of not having large, repeated regions, as is observed for Kaumoebaviruses and ASFV, but not for Faustoviruses. Of these 465 genes, about half (244 ORFs) encode for proteins with no hits in the nr database. Only one-third encode for proteins with a functional annotation. Moreover, a single tRNA (Ile-tRNA) also detected in six Asfarvirus MAGs, is present in the genomes of Pacmanvirus A23 and S19 [89]. The Pacmanvirus S19 genome is slightly longer with 418,588 bp and a similar GC content percent of 33.2%, which is lower than that of Asfarvirus, Kaumoebavirus, and Faustoviruses, which range from 37% to 43% [14,99]. Pacmanvirus lupus has a genome of 407,705 bp and probably represents a new Pacmanvirus lineage. The predicted major capsid protein in strain lupus is interrupted by at least two introns and a gene encoding a GIY-YIG endonuclease, as observed in Faustoviruses and Kaumoebaviruses, but which is not present in the two initial Pacmanvirus strains. A total of 31 functional co-orthologous genes are shared between Pacmanviruses, Asfarviruses, Faustoviruses, and Kaumoebaviruses. These core genes are, in particular, involved in DNA replication and damage repair [14]. Phylogenetic reconstruction based on the core gene of DNA polymerase gene shows that Pacmanviruses, ASFV, Faustoviruses, and Kaumoebavirus represent four distinct viral groups, clustering together, thus probably sharing a common ancestor. Kaumoebavirus roots these clusters, suggesting that the Kaumoebavirus group diverged before the other three viral groups.

### 5.4. Replication Cycle

The replication cycle of Pacmanvirus A23 has been exhaustively studied by transmission electron microscopy [14]. Pacmanvirus multiplies rapidly compared to Mimivirus, with a complete replication cycle of eight hours that ends with the complete lysis of its host *Acanthamoeba castellanii* for strain A23. The cycle starts with phagocytosis of the virions. Pacmanvirus seems to be able to escape the phagosomal step, as observed by a capsid opening that does not occur immediately. A few minutes after phagocytosis, once in the host’s cytoplasm, some virions migrate close to the mitochondria, but without membrane fusion. Although empty capsids have been detected in the cytoplasm, DNA release from the capsid has not been observed to date [14]. Early viral factories appear at three hours post-infection and typical viral factories at four hours post-infection. At this later time, neovirions can be visualised in the cytoplasm. After six hours, the amoeba cell is filled with new viral particles organised into geometric “honeycomb” forms or filling the entire host’s cytoplasm. Finally, cell lysis occurs at eight hours post-infection [14].

## 6. Abalone Asfarvirus

**Figure 1:**
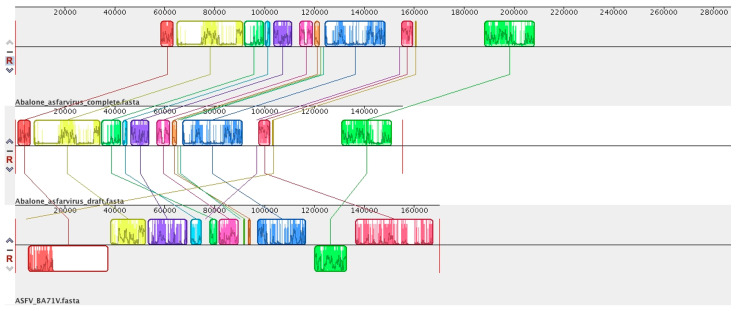
Genome synteny between Abalone Asfarvirus genome and the ASFV-BA71V genome. Schematic genome alignment obtained using the Mauve software [102]. The analysis was performed using the genome of Abalone Asfarvirus (LC637659.1), draft genome and ASFV strain BA71V (NC_001659.2). The blocks illustrated above the *x* axis are in the positive strand (forward sense), while blocks below the *x* axis are in the negative strand (reverse sense). The names of each virus are indicated below the sequence. The connected lines represent the relatively similar blocks between the genomes.

**Figure 2:**
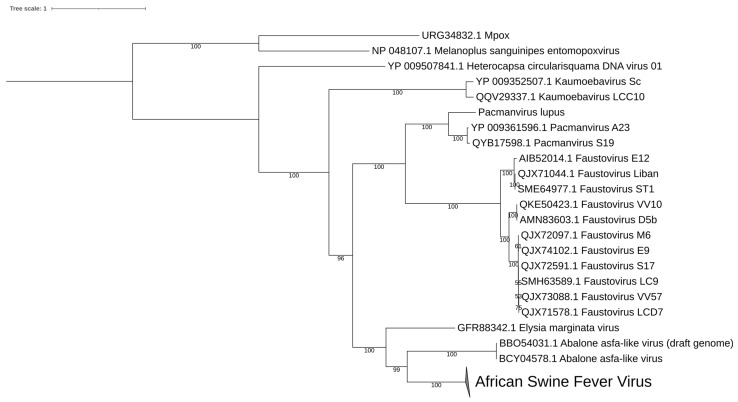
Phylogenetic tree based on the DNA polymerase homologs of *Asfarviridae* and relative viruses. ASFV contains 38 DNA polymerase sequences. Protein alignment was performed using Mafft software (v7.471) with standard parameters. The tree was built using IQ-TREE 1.6.12 with LG + F + I + G4 as best-fit model and 10,000 ultrafast bootstrap replication. *Poxviridae* sequences were used as an outgroup.

**Figure 3:**
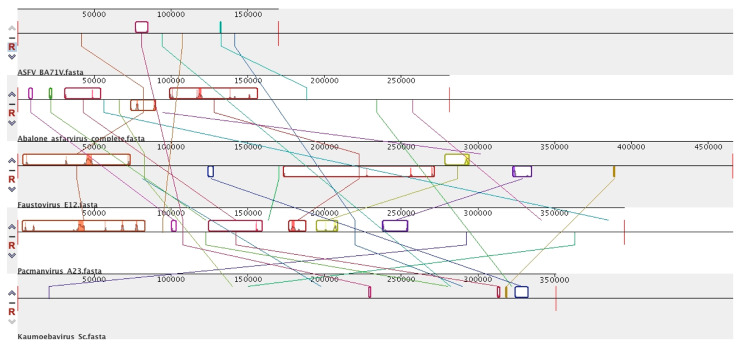
Mauve alignment genome of some *Asfarviridae* and other related viruses. Schematic genome alignment obtained using the Mauve software [102]. The analysis was performed using the genome of ASFV strain BA71V (NC_001659.2), Abalone Asfarvirus (LC637659.1), Faustovirus E12 (KJ614390.1), Pacmanvirus (LT706986.1) and Kaumoebavirus (KX552040.1). The blocks illustrated above the *x* axis are in the positive strand (forward sense), while blocks below the *x* axis are in the negative strand (reverse sense). Names of each virus are indicated below the sequence. The connected lines represent the relatively similar blocks between the genomes.

In 2020, a novel Asfarvirus-like virus, provisionally called Abalone asfar-like virus (AbALV), was identified as one causative agent of abalone amyotrophia, responsible for mass mortality in this gastropod mollusc [15]. Other viruses have been identified for amyotrophic disease, but the main difference consists in rapid onset, as is the case for While Abalone Herpesvirus (AbHV) and Abalone shrivelling syndrome-associated virus (AbSV). This amyotrophia seems to be related to this AbALV and causes mass mortality in juvenile abalone *Haliotis* spp. [101]. The initial molecular and immuno-staining experiments in laboratories appear to highlight a long period of incubation with a cumulative mortality increasing after 30 days post-infection, leading to the identification of a natural host resistance mechanism, as is the case in *Haliotis diversicolor*, and the virus has a preferential tropism for the gill [101]. This first prototype of AbALV virus had a draft linear genome of 151,181 bp (GC content = 31.63% NCBI: LC506465.1) predicted to encode 159 ORFs and no tRNA. The complete linear genome of AbALV strain MIE2018 is now available (21 September 2022) in the NCBI, with a larger genome of 281,224 bp (NCBI: LC637659.1) with 310 ORFs and no tRNA detected. Genomic comparison shows that AbALV is now complete, with the extension of both 5′ and 3′ ends of the genome (≈50,000 bp in both extremities) (Figure 1). These regions contain many hypothetical proteins with unknown functions. It can be noted that the genomic organisation is conserved between ASFV and the middle of the genome of AbALV, with a drastic reduction in ASFV genomes. Genomic and phylogenetic comparisons reveal that this virus is now the closest known relative of ASFV (Figure 1, Figure 2 and Figure 3). In addition, two ORFs encoding for a capsid homolog to p72 of ASFV were identified in AbALV genomes (BB054023.1/BCY004570.1 647 amino acids and BB054080.1/BCY04617.1 502 amino acids). No micrograph observations of this virus are currently available.

## 7. Novel Viral Detection by Metagenome-Assembled Genomes (MAG)

The expansion of next generation sequencing and metagenomic sequencing has resulted in numerous sequences becoming available in public databases. This has two major consequences: numerous mis-annotations using automatic annotation [103,104] particularly in NCLDV that classifying them mainly as bacteria; and the discovery of many viral homologs in eukaryote genomes, notably in fungi (as for *Phytophtora parasitica*) [105]. This viral presence was revealed by viral protein detection as in the project genome of *Elysia marginata* [106], an aquatic gastropod where, in some scaffolds, we detected some viral homologs and no evidence of viral genome integration in the candidate host DNA (NCBI: BMAT01005152.1). Thus, we are unaware whether this Asfarvirus-like is a contaminant in the seawater or a *bona fide* reflection of an *Elysia* spp. infection. Nevertheless, it could be very interesting to find expanded *Asfarviridae* sequences in gastropods, as its the case in abalone species for AbALV. In 2021, a study by Karki et al. [89] identified and detected 35 novel viral MAGs from mainly marine and freshwater metagenomes, with genome sizes ranging from 121 kbp to 580.8 kbp. Pangenome analysis detected a restricted core genome of 12 predicted proteins among 7410 orthologous groups.

## 8. Diversity in *Asfarviridae* and Relatives

As the latest pan-metagenomic analyses have shown, the number of shared proteins in this group is reduced to about ten among more than 7000 with an extreme diversity of genome size and genomic content [89]. Phylogenetic analysis on DNA polymerase conserved gene confirm this diversity among this group of virus (Figure 2) but points out that at the level of block organisation, the links are weaker (Figure 3). This variety is also expressed in the ultrastructural difference of these viruses as for the complex splicing capsid observed in Faustovirus and Kaumoebavirus, and with the multilayers and outer envelope observed in ASFVs in contrast to those described in Pacmanvirus.

## 9. Conclusions

Historically, ASFV was the only member of this *Asfarviridae* family. The recent isolation of these new giant viruses suggest a great diversity, which remains far from being completely elucidated. The intense economic interest in ASFV disease and the study of a vaccine candidate for pigs associated with the isolation and detection of these viruses in amoebae, gastropods and different marine metagenomes, illustrates the great advances made in recent years in *Asfarviridae*. Structural studies describe a complex capsid organisation for this group, expanding our knowledge of *Nucleocytoviricota* emphasising the separation between two sub-groups ASFV and abalone virus on the one hand and viruses from amoeba on the other.

## Figures and Tables

**Table 1:** Main identified virion proteins in ASFV and their homologs in other related viruses.

	Homologs/Virus	ASFV	Abalone Asfarvirus	Faustovirus	Pacmanvirus	Kaumoebavirus	Elysia Marginata
Outer envelope	pEP402R	Yes	No	No	No	No	No
MCP chaperone	B602L	Yes	Yes	Yes	Yes	No	Yes
Outer capsid	P72 capsid	Yes	Yes	Yes	Yes	Yes	Yes
	M1249L(mCP)	Yes	Yes	Yes	Yes	Yes	Yes
	P49	Yes	No	No	No	No	No
	H240R	Yes	Yes	No	No	No	Yes
Core proteic shell	pp220	Yes	Yes	Yes	Yes	Yes	yes
	pp62 (pp60)	Yes	Yes	Yes	Yes	Yes	yes
	pS273R	Yes	Yes	Yes	Yes	Yes	yes
Inner envelope	E199L	Yes	Yes	No	No	No	Yes
	p17	Yes	No	No	No	No	No
	p12	Yes	No	No	No	No	No
	E248R	Yes	Yes	No	Yes	Yes	Yes
	pE84R	Yes	No	No	No	No	No
Nucleoid	pK78R	Yes	No	No	No	No	No
	pA104R	Yes	Yes	No	No	No	Yes

“No” means proteins in ASFV that have no detected homologs by BLASTp. mCP: minor capsid protein.

## Data Availability

Not applicable.
